# Willingness to Pay for Cataract Surgery Provided by a Senior Surgeon in Urban Southern China

**DOI:** 10.1371/journal.pone.0142858

**Published:** 2015-11-17

**Authors:** Mei Wang, Yajing Zuo, Xianhua Lin, Yunlan Ling, Xiaofeng Lin, Mingge Li, Ecosse Lamoureux, Yingfeng Zheng

**Affiliations:** 1 Department of Ophthalmology, Sun Yat-sen Memorial Hospital, Sun Yat-sen University, Guangzhou, China; 2 State Key Laboratory of Ophthalmology, Zhongshan Ophthalmic Center, Sun Yat-sen University, Guangzhou, China; 3 Guangzhou Institutes of Biomedicine and Health, Chinese Academy of Sciences, Guangzhou, China; 4 Shantou Central Hospital, Affiliated Shantou Hospital of Sun Yat-sen University, Shantou, China; 5 Singapore Eye Research Institute, Singapore National Eye Centre, Singapore, Singapore; 6 Duke-National University of Singapore Graduate Medical School, Singapore, Singapore; 7 Centre for Eye Research Australia, University of Melbourne, Melbourne, Australia; Medical College of Soochow University, CHINA

## Abstract

**Purpose:**

To study willingness to pay for cataract surgery and surgical service provided by a senior cataract surgeon in urban Southern China.

**Methods:**

This study was a cross-sectional willingness-to-pay (WTP) interview using bidding formats. Two-hundred eleven persons with presenting visual impairment in either eye due to cataract were enrolled at a tertiary eye hospital. Participants underwent a comprehensive eye examination and a WTP interview for both surgery and service provided by a senior surgeon. Demographic information, socioeconomic status and clinical data were recorded.

**Results:**

Among 211 (98% response rate) persons completing the interview, 53.6% were women and 80.6% were retired. About 72.2% had a monthly income lower than 1000 renminbi (US $161). A total of 189 (89.6%) were willing to pay for cataract and the median amount of WTP was 6000 renminbi (US$968). And 102 (50.7%) were willing to pay additional fees for surgery performed by a senior surgeon, and the median amount of WTP was 500 renminbi (US$81). In regression models adjusting for age and gender, persons with preexisting eye diseases other than cataract, were more likely to pay for cataract surgery and service provided by a senior surgeon (P = 0.04 for both).

**Conclusions:**

In urban China, cataract patients, especially those with preexisting eye conditions, are willing to pay additional fees for a senior surgeon. Moving to a system where the price of cataract surgery is proportional to the consultant’ skill and expertise is possible and may have a potential impact on waiting list and quality of eye care. Further studies are needed to examine the impact of such pricing system on attitudes and choices of cataract patients.

## Introduction

Although blindness due to cataract is usually curable with an excellent prognosis for sight restoration, many patients with cataract do not receive medical treatment due to a variety of reasons including concerns of cost, fear of surgery, quality of services, transportation, and lack of family support.[1–5] Data from population-based studies show that only 3% of those aged 45+ years in North China,[6] and 4.4% of those aged 50+ in Urban Southern China underwent cataract surgery.[7] The World Health Organization (WHO) has set a cataract surgery rate (CSR, cataract operations per million population per year) of 3000 as the minimum necessary to eliminate cataract blindness, but China’s CSR is still lower than 1300.[8]

Existing cost system has a significant impact on patient’s choice and may eventually affect the quality and volume of cataract surgery. A recent population-based study shows that the 5-year incidence of cataract surgery in urban Southern China has improved to 4.8% in 2009,[9] probably due to improved health care access after the establishment of basic social medical insurance system (BSMIS). Efforts have been made to improve China’s health insurance coverage rate (over 95% across the nation)[10] and approximately 70% of medical fees are covered by health insurance,[11] and as a result, there have been some reports showing that cost may no longer be a major barrier to the uptake of surgery among the insured in urban China.[12]

However, the current price system of China’s price bureau may not be able to ensure that the quality of cataract surgery is being improved. Chinese patients pay similar amount of surgery fees regardless of surgeon’s professional level (although their reimbursement ratios are made differently, and senior surgeons usually have a higher pay than junior ones). Thus, a growing trend is that patients tend to seek treatments provided by senior consultants, because it doesn’t cost them more money to do so, and the already complex path of eye care is complicated by the fact that senior consultants have to squeeze in more patients at the cost of spending less time with each patient and less involvement in shared decision-making.[13;14] Patients with a higher socioeconomic status, especially those with a good social connection with clinicians, were more likely to receive faster service than those who do not. Many China’s provincial hospitals have been suffering from patient overcrowding, with families sleeping overnight on hospital lawns in an attempt to avoid early morning queues. In addition, junior consultants see much fewer cases per day and have fewer opportunities for practicing cataract surgery. It is therefore not a surprise to see that china has more than 23,000 ophthalmologists, but on average each doctor treats only approximately 24 cases of cataract per year.[15;16] These data confirm that eye services could have been better allocated between consumers.

One possible solution to this problem is perhaps a multi-tiered surgical pricing system that discourages patients from choosing expensive service provided by a senior surgeon when there are sufficient experienced young consultants who charge a lower price. This is based on the assumption that even modest costs can discourage patients from purchasing health care regardless of outcomes. It is unclear if patients would be willing to pay out-of-pocket for cataract procedures offered by senior surgeons, and how much they would like to pay. This study was designed to assess patient’s perception of surgery fees to senior surgeons who perform cataract surgeries. This study aims to provide information regarding these questions by obtaining data from a questionnaire survey of cataract patients in Urban Southern China.

## Methods

The study adhered to the Declaration of Helsinki and approvals were obtained from the Zhongshan Ophthalmic Center Institutional Review Board, and written informed consent was waived for the survey. The survey was explained with the notification that it was voluntary and would not influence care, and the data were analyzed anonymously.

### Study population

This study was a cross sectional questionnaire survey at the Second Affiliated Hospital, Sun Yat-sen University, Guangzhou, China between June 2013 and November 2013. All persons aged 18 years and older presenting to screening stations who had presenting visual impairment (visual acuity ≤0.5 in either eye) due to cataract were eligible to participate in the cataract study. Persons with previous cataract surgery in either eye were excluded. A comprehensive ophthalmic examination including presenting visual acuity, biomicroscopy, intraocular pressure, and funduscopy was provided. Blindness and severe visual impairment were classified according to the World Health Organization (WHO) criteria.

### Willingness to pay (WTP) survey

Interviewers who were trained by the investigating researchers administered the questionnaire survey. Patients were told that their responses were hypothetical and would not have any relationship with the hospital’s current services, provision of care or treatment costs; and they were also instructed that the hypothetical fees discussed should be considered as out-of-pocket expense that would potentially reduce the amount of money available for other household expenditures.

Willingness to pay for a standard cataract surgery performed by a trained surgeon, as well as willingness to pay additional fees for services offered by a senior cataract surgeon, were measured using a bidding format to stimulate a market situation, and prices were offered using randomly drawn payment cards to minimize starting point bias. The Prices were chosen based on estimated market price, and the starting points printed on the value cards were US$312.5, 781.25 or 1562.5 [2000, 5000 or 10000 renminbi (RMB)] for cataract surgery, and US$ 15.63, 78.13 or 156.25 [100, 500 or 1000 RMB] for a senior surgeon. A randomly drawn value card was shown to the patient, who was instructed to indicate that if s/he would or would not be willing to pay the amount printed on the card. If the answer was no, the interviewer dropped this amount at a specific interval (1000 RMB for cataract surgery; 100 RMB for services provided by a senior cataract surgeon) until the patient responded yes. If the answer to the initial amount was yes, the interviewer increased the amount until the patient was unwilling to pay. At the end of the survey, the interviewer repeated and confirmed the final amount with the patient. If the patients responded that they were unwilling to pay, the figure was recorded as zero and the reason for unwilling to pay was noted.

Following determination of willing to pay, demographic details were collected by questionnaire, and these included age, sex, patients’ past medical history, job type, employment, health insurance, educational level and annual income.

### Statistical analysis

The distribution of outcome variable in this study was skewed, and data were expressed as median and interquartile range (IQR). The Chi-square test or Fisher exact probability was applied for comparison of those willing and unwilling to pay. A 2-tailed *P* value <0.05 was considered statistically significant. Quantitative data were analyzed using SPSS statistical software version 20.0 (SPSS Inc, Chicago, IL, USA).

## Results

Among 215 eligible patients, 211 agreed (98% response rate). The median age was 73 years (IQR, 20–85), with 53.6% being female and 80.6% being retired. About 72.2% of subjects had a monthly income lower than 1000RMB (US $161). A total of 205 participants had health insurance, 82.3% of them had government health insurance whereas 9.3% had private health insurance.

Among the 211 participants, 189 (89.6%) were willing to pay for cataract surgery. The median amount these subjects were willing to pay was 6000 RMB (US $ 968)(IQR, 1000- 10000RMB or US $161–1614). There were no significant differences in the WTP among subjects of all income levels. Average annual per capita incomes of rural households in 2003 were 2622 RMB in China and 4055 RMB in Guangdong Province.

Among those unwilling to pay anything for surgery, the most common reasons given were “not enough income” (15/22; 68.2%), waiting to have a free surgical service covered by the government (4/22; 18.2%) or by non-government organization (NGO) (2/22; 9.1%), and that s/he did not believe that surgery could improve vision (1/22; 4.5%) of those unwilling to pay for surgery thought that surgery will not improve vision.

There were no significant differences in baseline characteristics such as age, gender, visual acuity in the operated eye, job type, employment, and systemic conditions between those who were willing and those who were unwilling to pay for cataract surgery. Those who were unwilling to pay anything for cataract surgery were more likely to be the ones with lower income levels, without health insurance and preexisting eye diseases other than cataract (p>0.05 for all) (Table 1). In the multivariate analysis adjusting for age and gender, preexisting eye diseases remained a significant factor associating with the willingness to pay for cataract surgery (p = 0.04).

**Table 1:** Analysis of willing to pay for cataract surgery, Guangzhou, China.

Characteristics	Subgroups	Willing to pay for cataract surgery		p-value#
		No	Yes	
Age				0.428
	<70	10 (50)	73 (40.8)	
	≥70	10 (50)	106 (59.2)	
Gender				0.421
	Female	10 (45.5)	103 (54.5)	
	Male	12 (54.5)	86 (45.5)	
Presenting Visual acuity				0.062
	<0.3	17 (81.0)	172 (94.5)	
	≥0.3	4 (19.0)	10 (5.5)	
Monthly income (RMB*)				**0.014**
	<1000	20 (90.9)	123 (65.1)	
	≥1000	2 (9.1)	66 (34.9)	
Main employer				0.095
	Public sector	14 (63.6)	84 (44.4)	
	Private sector or Self-employed	1 (4.5)	43 (22.8)	
	Informal employment	7 (31.8)	62 (32.8)	
Working state				0.515
	Retired	20 (90.9)	150 (79.4)	
	Still working	0 (0)	14 (7.4)	
	Unemployed	2 (9.1)	25 (13.2)	
Health insurance				**0.044**
	Basic medical insurance	14 (66.7)	155 (84.2)	
	Free medical service	5 (23.8)	14 (7.6)	
	Commercial medical insurance	2 (9.5)	15 (8.2)	
Eye disease other than cataract				**0.030**
	Yes	2 (9.1)	59 (31.2)	
	No	20 (90.9)	130 (68.8)	
Systemic diseases				0.551
	Yes	9 (40.9)	90 (47.6)	
	No	13 (59.1)	99 (52.4)	

# Chi-square test

* 6.1976 RMB = 1 US dollar (1 RMB = US $0.1614)

Slightly more than half (50.7%) of the subjects said that they would be willing to pay an additional fee to have the surgery performed by one of the senior doctors. The median amount that these subjects were willing to pay was 500 RMB (US $ 81) (IQR, 100–2000 RMB or US $16–161).

The major cause of unwillingness to pay an additional fee for surgery performed by a senior doctor was that s/he was comfortable with the surgery performed by a trained surgeon (85/100; 85%). The rest felt that they could not afford to do so (15/100; 15%).

In the multivariate analysis investigating potential association with the willingness to pay for an additional fees for surgical service provided by a senior surgeon, participants with preexisting eye diseases other than cataract were more likely to be the ones who were willing to pay an additional fee for such a service enhancement (p = 0.05). Other characteristics such as age, gender, visual acuity, income, job type, health insurance, and having systemic disease were not associated with willing to pay (Table 2).

**Table 2:** Analysis of willing to pay an additional fee for surgery performed by a senior surgeon, Guangzhou, China.

Characteristics	Subgroups	Willing to pay an additional fee for one of the senior doctors		p-value#
		No	Yes	
Age				0.895
	<70 years	40 (41.2)	43 (42.2)	
	≥70 years	57 (58.8)	59 (57.8)	
Gender				0.719
	Female	57 (54.8)	56 (52.8)	
	Male	47 (45.2)	51 (48.1)	
Presenting Visual acuity				0.244
	<0.3	91 (91)	98 (95.1)	
	≥0.3	9 (9)	5 (4.9)	
Monthly income (RMB*)				0.183
	<1000	75 (72.1)	68 (63.6)	
	≥1000	29 (27.9)	39 (36.5)	
Main employer				0.957
	Public sector	49 (47.1)	49 (45.8)	
	Private sector or Self-employed	22 (21.2)	22 (20.6)	
	Informal employment	33 (31.7)	36 (33.6)	
Working state				0.869
	Retired	85 (81.7)	85 (79.4)	
	Still working	6 (5.8)	8 (7.5)	
	Unemployed	13 (12.5)	14 (13.1)	
Health insurance				0.792
	Basic medical insurance	84 (84)	85 (81)	
	Free medical service	9 (9)	10 (9.5)	
	Commercial medical insurance	7 (7)	10 (9.5)	
Eye disease other than cataract				**0.032**
	Yes	23 (22.1)	38 (35.5)	
	No	81 (77.9)	69 (64.5)	
Systemic diseases				0.440
	Yes	46 (44.2)	53 (49.5)	
	No	58 (55.8)	54 (50.5)	

# Chi-square test

* 6.1976 RMB = 1 US dollar (1 RMB = US $0.1614)

## Discussion

As concerns about rising eye care costs continue to grow, there is an increasing interest among policy makers and payers regarding how to design innovative payment mechanism and pricing program that give patients more accountability for the costs associated with their care. Ideally, the pricing structures for cataract surgery need to be flexible, with different levels, to improve affordability and increase access for those at lower incomes. The pricing system should also take into account the efficiency and skill of cataract surgeons and how the senior surgeons should be reimbursed. It is important to understand the perceived value of specific types of treatment to patients, and this could benefit from WTP data. To the best of our knowledge, most of the WTP surveys have been conducted in rural areas,[17–21] and this study is the first to document willingness to pay for cataract surgery and for potential enhancement of cataract surgical service provided by a senior surgeon among participants living in urban area.

Not surprisingly, around 89.6% of participants were willing to pay for cataract surgery. This value is higher than reported in rural regions in China (80%),[17] Nepal (56.4%),19 and Tanzania (60%),[18] and the discrepancy probably reflects differences in knowledge, attitudes, and affordability of participants with different socioeconomic levels. The total WTP for cataract surgery was 6000 RMB (US$968), which is lower than the real average total cost (7400 RMB, or US$1193) for one cataract surgery episode in a tertiary urban hospital in Southern China.[17] It may be that patients would be willing to pay less if faced with the need to pay out-of-pocket for their eye care treatment. The WTP for cataract surgery in this urban region is much higher than that in rural Southern China. In Yangjiang and several other rural counties in Guangdong Province, for example, the cost of cataract surgery was set at 500 RMB (US$62.5).[20] A distinction must be made that the patients in urban cities usually receive phacoemulsification with an imported intra-ocular lens (IOLs), whereas the 500-RMB-surgery in rural area was usually refereed to manual small-incision cataract surgery (SICS), which is less expensive and less technology-dependent than phacoemulsification, yet it could achieve excellent visual outcomes and low complication rate.[22]

About half (50.7%) of the subjects said that they would be willing to pay an additional fee for senior surgeon. The median amount of WTP was 500 RMB (US$ 81), half of minimum monthly wage in Guangzhou city (1300 RMB or US$210). This is higher that the figure from Yangjiang, a rural county in Guangdong province.[20] In the Yangjiang study, the proportion of participants who were willing to additionally pay for senior surgeons was 36%, and the mean WTP amount was 175 RMB (US$18.3).[20] The difference in WTP between the two regions is explicable on the ground that the monthly per capita income in Guangzhou was 1.72 times than those in Yangjiang (5808 versus 3365 RMB) (Bureau of Statistics of Guangdong Province 2013). These findings suggest that quality of cataract surgical services remains a major concern in both urban and rural areas of China, although previous studies have indicated that trained surgeons can achieve excellent surgical outcomes with low complication rates. Our data have significant implications in implementing clinician prices in China. To keep up with its soaring demand for quality health care, China is undergoing substantial reforms to improve efficiency and quality of care, and Chinese authorities have been embracing innovative ideas to improve the health system. Given that around 50% of patients would be willing to pay for an enhancement of service provided by a senior surgeon, a multi-tiered surgical pricing framework that appropriately price surgeon’s skill and experience may be desirable in China, and could potentially reduce stress and increase efficiency among senior surgeons. Given more time and paid more, one would expect that the surgeons, both from public and private sectors, would communicate better with patients, a change that might improve patients’ satisfactory level.

Consistent with the previous report in Yangjiang city,[20] our study did not detect the differences in WTP for a senior surgeon between male and female patients and between high- and low-income groups. Interestingly, having preexisting eye diseases other than cataract had the strongest influence on WTP for a senior doctor, suggesting that those with preexisting eye conditions are better informed and place a higher value than patients with cataract only on physician’s technical skills and experience.

There were several limitations to our study. Our population was hospital-based and the surveyed participants might not be representative of the general population, but the WTP data from these persons are more relevant to our price system for those presenting for eye care in urban hospitals. In addition, as people value quality in different ways, we did not ask patients directly what criteria they used to define “senior surgeon”; it may be that the WTP surveyed in our study are not what drive the choice of a senior surgeon.

In conclusion, this study reveals a WTP for cataract surgery and service provided by a senior surgeon among urban Chinese patients. Our study suggests the possibility of an 8% increase in cataract surgical fees. Patients having preexisting eye diseases other than cataract may have a higher expectation for care from their cataract surgeons. If patients are willing to pay a greater share of costs, a pricing system that allows surgeon fees to be adjusted for factors such as technical skills and experiment may be valid. The government controlled surgical pricing system may not be properly aligned with the realities of eye care delivery. Future research should aim to further delineate feasibility and possible benefit of multi-tier surgical fees and investigate whether this would lead to shorter waiting time, better patient-physician communication, more productive visits, and a higher surgery volume among junior surgeons.

## Supporting Information

S1 Dataset Spreadsheet containing the data used in this study. (XLSX)
